# Candidate miRNA Regulators of Blood Transcriptional Signatures for Differential Diagnosis of Chronic Lymphocytic Leukemia and Multiple Myeloma: A Comprehensive In Silico Study

**DOI:** 10.3390/cimb48040352

**Published:** 2026-03-27

**Authors:** Gözde Öztan, Halim İşsever, Tuğçe İşsever

**Affiliations:** 1 Department of Medical Biology, Istanbul Faculty of Medicine, Istanbul University, Topkapı, 34093 Istanbul, Turkey; 2 Department of Public Health, Istanbul Faculty of Medicine, Istanbul University, Topkapı, 34093 Istanbul, Turkey; hissever@istanbul.edu.tr; 3 Turkish Health Institutes Presidency (TUSEB), 34718 Istanbul, Turkey; tugceissever@gmail.com

**Keywords:** chronic lymphocytic leukemia, multiple myeloma, miRNA regulators, biomarkers

## Abstract

Chronic lymphocytic leukemia (CLL) and multiple myeloma (MM) are biologically distinct hematologic malignancies with heterogeneous clinical courses, and minimally invasive molecular biomarkers are needed to support blood-based discrimination. We performed a comprehensive in silico analysis to derive cross-cohort, direction-consistent transcriptomic programs for CLL and MM and to nominate regulatory microRNAs (miRNAs) linked to these signatures. Public gene-expression datasets from the NCBI Gene Expression Omnibus (two cohorts per disease) were processed with a reproducible workflow to define disease-biased consensus gene sets. Experimentally validated miRNA–target interactions from miRTarBase were integrated with consensus genes for miRNA target over-representation analysis, and miRNA–mRNA networks were constructed to prioritize candidate miRNAs by connectivity. A strict intersection strategy yielded a large, direction-consistent CLL consensus program, whereas a vote-based approach produced a smaller MM program due to a weaker signal in one cohort. Enrichment and network analyses identified compact regulatory modules in CLL, including a highly connected candidate miRNA linked to many CLL-up genes. This framework provides reproducible disease-biased gene programs and evidence-anchored miRNA candidates to support targeted experimental validation and the development of hypothesis-driven blood-based biomarker studies for differential diagnosis and monitoring.

## 1. Introduction

Cancer remains a leading cause of morbidity and mortality worldwide, with the 2022 global estimates indicating roughly 20 million new cases and 9.7 million deaths, underscoring a persistent need for improved prevention, detection, and treatment strategies [1]. Within hematologic malignancies, chronic lymphocytic leukemia (CLL) and multiple myeloma (MM) represent two major entities with distinct cellular origins and clinical trajectories, yet both pose ongoing challenges related to biological heterogeneity and longitudinal disease monitoring [2]. Population-based analyses continue to demonstrate a substantial burden of both CLL and MM, as reflected in contemporary incidence and mortality patterns and long-term trends reported in registry studies and global burden analyses [3].

In current clinical practice, CLL diagnosis and risk stratification rely on a multi-component framework incorporating persistent peripheral blood B-lymphocytosis, immunophenotyping to establish clonality, and integration of prognostic biomarkers and response criteria [2]. In MM, diagnosis classically integrates bone marrow plasma cell assessment, monoclonal protein measurements, and evaluation for myeloma-defining events, with the IMWG criteria also incorporating validated biomarkers that predict near-inevitable progression in high-risk states [4]. Despite therapeutic advances, both diseases frequently require repeated evaluations over time, and the increasing emphasis on sensitive response assessment (including MRD-oriented concepts in CLL) strengthens the rationale for minimally invasive, standardized molecular markers that can complement conventional testing [5].

MicroRNAs (miRNAs) are ~22-nucleotide endogenous non-coding RNAs that regulate gene expression post-transcriptionally and can exert broad biological effects by coordinately modulating multiple targets and pathways [6]. Beyond their intracellular regulatory roles, miRNAs are detectable in circulation, including within extracellular vesicles (e.g., exosomes) or in association with protein complexes, which supports their evaluation as liquid-biopsy-style biomarkers. Seminal experimental work demonstrated that miRNAs can be present in plasma in a remarkably stable form, providing a mechanistic basis for their measurement in blood-based assays [7]. At the same time, translation of circulating miRNA biomarkers requires stringent control of pre-analytical variability (e.g., hemolysis, cellular contamination, processing and storage conditions) and harmonized analytical/normalization strategies to ensure reproducibility across cohorts and laboratories. Methodological syntheses further emphasize that while circulating miRNAs can be stable, specific miRNAs and measurement platforms may differ in resilience and susceptibility to technical confounders, reinforcing the need for standardized workflows [8]. If future follow-up studies translate top candidates into qPCR-based assays, adherence to the MIQE reporting guidelines would improve analytical transparency and cross-laboratory reproducibility [9]. Given the importance of circulating miRNAs and the emerging role of exosomes as vehicles of intercellular communication, it is also relevant to consider how alterations in exosomal miRNA cargo and exosome-mediated signaling may reflect or contribute to the pathological processes underlying CLL and MM [7,10]

In CLL, miRNA dysregulation is a well-established regulatory layer linked to disease biology and clinical behavior [11]. A landmark observation in CLL is the localization of miR-15 and miR-16 at chromosome 13q14, a region frequently deleted in CLL, which helped establish a mechanistic paradigm connecting non-coding RNA alterations to malignant phenotypes [12]. Subsequent work has further clarified functional roles of the miR-15a/miR-16-1 cluster in leukemia-relevant pathways, supporting its continued relevance in CLL pathogenesis and biomarker frameworks [13]. In parallel, studies measuring circulating miRNAs in CLL have suggested that plasma/serum miRNA levels can associate with clinical states and treatment responses, illustrating the feasibility of blood-based miRNA monitoring in this disease context [14].

MM is strongly shaped by the bone marrow microenvironment, and extracellular vesicle-mediated communication has emerged as a biologically plausible layer through which miRNA cargo may reflect disease activity and progression. Recent reviews and translational studies highlight that exosomal miRNAs are being actively investigated as candidate biomarkers for MM and related monoclonal gammopathies, although broader validation across independent cohorts remains essential [10]. Mechanistic evidence also supports interpretability of miRNA candidates in MM-relevant survival signaling, as miR-24-3p has been linked to IL-6–dependent plasma cell survival, connecting extracellular cues to intracellular miRNA-mediated regulation [15].

Given the heterogeneity of both CLL and MM, single-cohort findings can be difficult to generalize, motivating integrative analyses that combine multiple independent datasets with evidence-anchored regulatory inference [8]. Public repositories such as NCBI GEO provide MIAME-compliant access to transcriptomic datasets and support transparent, reusable workflows for cross-cohort discovery and benchmarking. GEO was originally established to archive and distribute high-throughput gene expression data with accessioned records and accompanying metadata, enabling secondary analyses and reuse across studies [16]. The MIAME standard specifies the minimum information needed to interpret and verify microarray experiments, thereby supporting meaningful deposition and reproducibility in public repositories [17]. For miRNA-level interpretation, prioritizing candidates using experimentally validated miRNA–target interactions strengthens the evidentiary basis beyond prediction-only approaches, and miRTarBase represents a major curated resource for such validated interactions [18].

To further enhance interpretability, disease-biased gene programs can be contextualized at the network level by constructing miRNA–mRNA bipartite regulatory networks and prioritizing hub miRNAs using topology-based metrics, while enrichment tools such as Enrichr facilitate pathway- and ontology-based annotation of the resulting programs [19]. Accordingly, our study aims to connect cross-cohort transcriptomic signatures to high-confidence miRNA regulators by integrating GEO-derived gene programs with miRTarBase-supported interactions and network/topology-driven prioritization, thereby yielding reproducible candidate shortlists and mechanistically interpretable hub networks for downstream experimental validation and future classifier benchmarking rather than direct diagnostic performance estimation in the present study.

## 2. Materials and Methods

### 2.1. Study Design and Analytical Workflow

We implemented an integrative, evidence-constrained analytical framework to link cross-cohort gene expression patterns with validated miRNA-target interactions, enabling topology-informed prioritization of candidate hub regulators and generation of reproducible candidate sets for downstream experimental validation. The workflow comprised GEO dataset selection, standardized processing of differential-expression summary tables, volcano-plot visualization with harmonized thresholds, extraction of top-ranked upregulated and downregulated genes per dataset, and cross-dataset consensus analysis to derive disease-specific and shared gene signatures for CLL and MM.

### 2.2. Data Source and Dataset Selection

All datasets were obtained from the NCBI Gene Expression Omnibus (GEO) database [16]. Datasets were selected to represent human samples with clearly defined disease and healthy control groups and to be compatible with a PBMC/whole blood–oriented framework. Datasets were included if they met the following criteria: human disease and healthy control samples, accessible processed differential-expression outputs or analyzable GEO-derived summaries, compatible blood- or PBMC-oriented sampling context, and sufficient annotation to support cross-cohort comparison. Datasets lacking an interpretable case–control structure, adequate annotation, or compatibility with the comparative framework were excluded. Two independent datasets were analyzed for each condition to support cross-cohort reproducibility. For CLL, GSE50006 and GSE31048 were included. For MM, GSE7116 and GSE27838 were included; for GSE27838, the “non-expanded” subset was used for MM and healthy controls to maintain a consistent comparison framework. The principal characteristics of the included GEO cohorts are summarized in Table 1.

### 2.3. Differential Expression Summary Inputs and Preprocessing

For each dataset, differential expression results were analyzed using “top.table” style summary files that included gene annotations and statistical outputs. Because all included cohorts were microarray-based GEO datasets, differential expression was not re-computed from raw CEL files or other raw expression matrices; instead, downstream analyses were performed using the available processed top-table summaries (log2FC and BH-FDR) to preserve consistency with the original study-specific preprocessing pipelines. Required fields were gene symbol (or an equivalent gene annotation), log2 fold-change (log2FC), and FDR-adjusted *p*-values. Where available, nominal *p*-values and additional annotation fields were retained for reporting. All summary tables were imported into a unified processing pipeline and standardized to a common schema to enable harmonized filtering, ranking, and visualization. Accordingly, the present study should be interpreted as a cross-cohort summary-analysis framework rather than a uniform raw-data reprocessing or formal meta-analysis pipeline. All downstream comparative analyses were therefore performed on harmonized differential-expression summary outputs rather than uniformly reprocessed raw expression matrices. Consequently, formal cell-type deconvolution was not implemented in the present study. Because the included GEO cohorts were generated on different microarray platforms and processed under study-specific normalization workflows, residual technical heterogeneity cannot be excluded. The present analysis sought to mitigate this by applying harmonized downstream filtering, direction-aware consensus construction, and cross-cohort comparison at the level of processed differential-expression summaries rather than raw-matrix recomputation.

### 2.4. Gene Symbol Curation and Filtering

To avoid platform-specific artifacts and improve interpretability, gene symbols were curated prior to top-gene labeling and consensus analyses. Probe-like identifiers and non-gene labels were excluded, including microarray probe IDs ending in “_at” (for example, “238604_at”). Composite gene labels containing multiple mappings were cleaned by retaining a single representative symbol when feasible; unresolved entries were excluded from top-gene reporting. Duplicate gene symbols were removed by retaining the entry with the smallest FDR (and the largest absolute log2FC in case of ties), ensuring that each gene was represented only once in the ranked outputs and annotations. Annotated LOC entries (“LOC”, i.e., locus-based provisional gene annotations), pseudogenes, and non-coding loci were retained in ranked reporting when they represented valid unique annotations in the processed source tables; however, these entries were interpreted cautiously because they may be less transferable to downstream functional or clinical validation than well-characterized protein-coding genes.

### 2.5. Statistical Thresholds and Volcano Plot Visualization

Volcano plots were generated for each dataset using log2FC (disease vs. healthy) on the *x*-axis and −log10 (FDR-adjusted *p* value) on the *y*-axis. Harmonized primary thresholds were set at FDR < 0.05 and |log2FC| ≥ 1, and these cutoffs were displayed as dashed horizontal and vertical reference lines. Significant genes were highlighted by direction of change. In low-signal cohorts (e.g., GSE27838), gene labels were applied based on FDR-ranked top genes after symbol filtering, even when not all labeled genes met the FDR < 0.05 criterion, to support structured hypothesis generation while preserving the standard threshold lines for interpretability. Although alternative threshold settings were not systematically benchmarked in the present version, future sensitivity analyses should test the stability of consensus signatures across varying FDR and effect-size cutoffs.

### 2.6. Selection of Top-Ranked Genes and Generation of Reporting Tables

For each dataset, the top 10 upregulated and top 10 downregulated genes were selected for reporting based on adjusted significance ranking, after applying gene symbol filtering and de-duplication. This reporting step was applied uniformly across cohorts; therefore, in datasets with few FDR-significant genes under the harmonized thresholds (e.g., GSE27838), top-ranked genes should be interpreted as prioritized candidates rather than definitive FDR-positive biomarkers. For each cohort, a structured summary table was generated reporting gene symbols, log2FC, nominal *p*-values (if available), and FDR. Tables were exported as excel files with separate sheets for upregulated genes, downregulated genes, and a combined top-20 list. This top-10 reporting step was used for standardized visualization and summary reporting only and did not define the downstream consensus signatures, enrichment analyses, or network construction, which were based on the broader processed differential-expression outputs and derived consensus gene sets.

### 2.7. Cross-Dataset Consensus Signature Analysis

Consensus gene signatures were derived separately for CLL and MM, incorporating directionality to ensure biological consistency. For CLL, a strict intersection approach was applied using GSE50006 and GSE31048: genes were retained in the CLL-consensus set if they were significant under the predefined thresholds in both datasets and showed concordant direction of change. For MM, a vote-based consensus approach was used because GSE27838 yielded limited genes under strict multiple-testing thresholds; therefore, genes were retained in the MM-consensus set if they showed concordant direction across GSE7116 and GSE27838, met the absolute fold-change threshold in both datasets, and were statistically supported in at least one dataset. This asymmetric design was chosen because the CLL cohorts retained a large, direction-consistent overlap under strict thresholds, whereas the MM cohorts showed markedly reduced overlap when strict intersection was imposed. The vote-based MM rule was therefore used as a conservative compromise to preserve direction-consistent candidates while limiting dependence on a single underpowered cohort. Formal robustness analyses of consensus stability under alternative voting rules were not implemented in the present version and remain a future extension.

### 2.8. Definition of Disease-Specific and Shared Signatures

To support differential diagnosis between CLL and MM, three gene sets were derived using set operations applied to the CLL-consensus and MM-consensus lists. CLL-specific genes were defined as genes present in the CLL-consensus set but absent from the MM-consensus set. MM-specific genes were defined as genes present in the MM-consensus set but absent from the CLL-consensus set. Shared genes were defined as the intersection between the CLL-consensus and MM-consensus sets. All three lists preserved directionality and included summary statistics required for downstream interpretation.

### 2.9. Preparation of Input Gene Sets for miRNA Enrichment

The miRNA enrichment analysis was performed using the disease-specific and shared consensus gene signatures derived from the cross-dataset consensus step. Three primary gene sets were used as inputs: the CLL-specific signature, the MM-specific signature, and the shared signature. To preserve regulatory interpretability, each signature was further stratified by direction of change (up and down), based on the directionality assigned during consensus construction. Before enrichment, all input lists were checked for valid HGNC-style gene symbols, and any remaining ambiguous or non-standard identifiers were removed. The final input sets included CLL-specific up, CLL-specific down, MM-specific up, MM-specific down, and shared genes, enabling direction-aware inference of upstream miRNA regulators. When a direction-stratified input set contained too few genes to support stable inference (e.g., MM-specific up or concordant shared subsets), enrichment results were reported primarily for the direction-aware sets with adequate size and diagnostic interpretability (CLL-specific up/down, MM-specific down, and discordant shared CLL up/MM down).

### 2.10. miRNA–Target Knowledge Base and Target Universe Definition

To infer candidate miRNA regulators associated with disease-related blood transcriptional programs, validated miRNA–target interactions were compiled from curated miRNA resources. In this study, “miRNA–target” is used as the broader term for curated regulatory interactions, whereas “miRNA–mRNA” refers specifically to cases in which the target is represented as a protein-coding gene transcript in enrichment analyses or network visualizations. Experimentally validated interactions were prioritized using miRTarBase [18]. MiRTarBase-derived “microRNA Targets” gene set libraries were accessed via Harmonizome [20] to obtain enrichment-ready target sets. This curated evidence layer was prioritized to reduce false-positive target inflation. Accordingly, miRNA–target pairs were filtered to retain experimentally supported interactions whenever possible, and the target universe for enrichment was defined as the set of all unique, valid gene symbols present in the processed differential-expression tables across the four included GEO datasets. These interactions were restricted to experimentally validated miRNA–target relationships curated in miRTarBase-derived resources; however, no additional stratification by evidence subtype or assay strength was imposed in the present network construction. The total size of this background universe was *n* = 21,755 unique valid gene symbols across the four processed datasets. Using a dataset-specific target universe helps minimize background bias caused by platform coverage differences and improves comparability across CLL and MM cohorts. Alternative background definitions, including platform-specific measured-gene sets or all tested genes per cohort, may influence enrichment statistics and should be examined in future robustness analyses.

### 2.11. miRNA Enrichment Analysis and Statistical Testing

miRNA enrichment was conducted as an over-representation analysis to test whether the genes in each input signature were significantly enriched among the known targets of specific miRNAs. For each miRNA, a 2 × 2 contingency table was constructed using the signature gene set and the defined target universe, and enrichment significance was evaluated using a hypergeometric test (equivalently Fisher’s exact test). Multiple testing correction was performed using the Benjamini–Hochberg procedure, and miRNAs were considered significantly enriched based on an FDR-adjusted *p*-value threshold. For each signature, enriched miRNAs were ranked by adjusted significance and effect size metrics, including the number of overlapping targets and enrichment ratio. No hard minimum overlap threshold was imposed in the present version; therefore, enriched miRNAs associated with very small overlap counts or very small underlying target sets were interpreted cautiously, particularly in MM-focused analyses. Future refinements may incorporate explicit minimum overlap thresholds and/or minimum regulon-size filters to improve biological credibility. Analyses were performed separately for CLL-specific up/down, MM-specific up/down, and the shared signature to identify disease-biased regulatory candidates. All statistical computations were implemented using in-house scripts in R (version 4.5.2, R Foundation for Statistical Computing, Vienna, Austria) and Python (version 3.14.2, Python Software Foundation, Wilmington, DE, USA).

### 2.12. Prioritization of Differential-Diagnosis Candidate miRNAs (CLL vs. MM)

To support differential diagnosis, enriched miRNAs were categorized into three groups: CLL-biased, MM-biased, and shared miRNAs. A miRNA was considered CLL-biased if it was significantly enriched in at least one CLL-specific signature (up or down) and not significantly enriched in MM-specific signatures, while MM-biased miRNAs were defined analogously. miRNAs enriched in both diseases or primarily in the shared gene set were classified as shared candidates and interpreted as reflecting common hematologic or immune-related regulation rather than disease specificity. In addition, a composite prioritization score was computed for each miRNA by integrating adjusted significance, overlap size (number of targeted genes within the signature), and consistency across direction-stratified gene sets. The top-ranked miRNAs from each disease-biased category were selected for downstream network construction and literature-supported interpretation.

### 2.13. Construction of miRNA–mRNA Regulatory Networks and Hub Identification

Bipartite miRNA–mRNA networks were built for CLL-specific and MM-specific signatures using enriched miRNAs and the overlapping protein-coding target genes derived from validated miRNA–target interaction sets. Network edges represented curated miRNA–target interactions, and node attributes included direction of gene dysregulation, consensus statistics, and enrichment metrics. Network topology was evaluated to identify hub miRNAs and hub target genes using degree-based hub ranking. Network visualization and inspection were performed in Cytoscape (version 3.10.4, Cytoscape Consortium, San Diego, CA, USA) [21]. Cytoscape is a widely used software environment for integrated visualization and analysis of biomolecular interaction networks, supporting network-centric interpretation in omics studies. Disease-specific networks were analyzed independently and then compared to determine regulatory structures that may differentiate CLL from MM.

### 2.14. Reporting Outputs and Visualization for miRNA Enrichment

For each signature, the enrichment results were reported as ranked tables including miRNA name, adjusted *p*-value, enrichment ratio, and the list and count of overlapping targets. Visualization outputs were generated to summarize the results and support interpretability, including bar plots of the top enriched miRNAs for each signature, Venn or UpSet-style overlap summaries for CLL-biased and MM-biased miRNAs, and network visualizations of the highest-confidence miRNA–mRNA modules derived from validated miRNA–target interactions.

## 3. Results

### 3.1. Study Selection and Dataset Characteristics for CLL (GSE50006, GSE31048)

Two independent GEO cohorts were analyzed to define a robust transcriptional signature of CLL. For GSE31048, the cohort included 179 CLL samples and 33 healthy controls, enabling higher-powered differential expression inference. For GSE50006, CLL and healthy groups were analyzed in the same manner to provide an independent validation cohort.

To ensure interpretability and avoid redundancy in downstream biomarker and network analyses, we applied a harmonized filtering strategy across datasets: probe-like identifiers (e.g., “*_at”) and non-gene labels were excluded, multi-mapped labels were cleaned to retain a valid gene symbol when possible, and duplicate gene symbols were removed so that each gene was represented once in ranked lists and annotations. Differential expression results were summarized using volcano plots (log2FC vs. −log10FDR), and the top 10 upregulated and top 10 downregulated genes were highlighted for each cohort.

#### 3.1.1. Differential Gene Expression Profile in CLL: GSE50006

**Figure 1:**
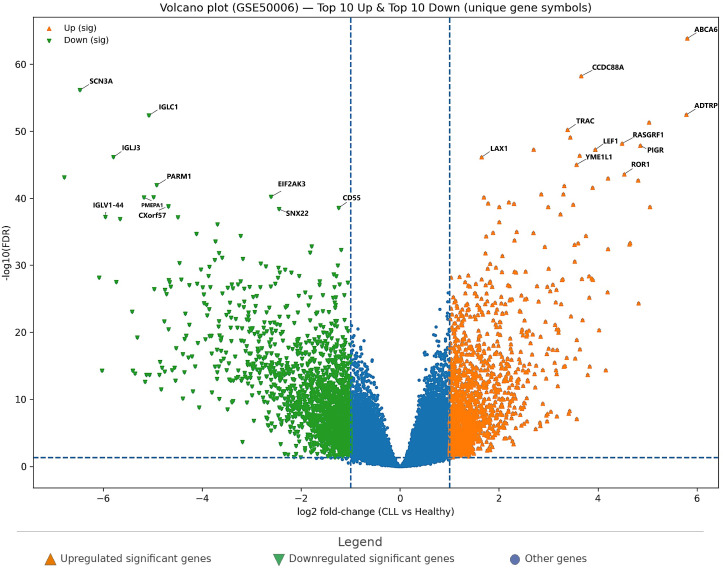
Volcano plot of differential gene expression in GSE50006 (CLL vs. healthy controls). Genes are plotted by log2 fold-change on the *x*-axis and −log10(FDR) on the *y*-axis. Dashed vertical lines indicate the fold-change threshold, and the dashed horizontal line indicates the significance threshold. Orange triangles indicate significantly upregulated genes, and green inverted triangles indicate significantly downregulated genes. Labeled points represent the top-ranked upregulated and downregulated genes selected for downstream comparative analysis.

In GSE50006, the volcano plot (Figure 1) shows a clear separation of significantly dysregulated genes between CLL and healthy controls. Using the thresholds FDR < 0.05 and |log2FC| ≥ 1, a total of 3123 significant genes were identified, comprising 1578 upregulated and 1545 downregulated genes. This balanced up/down distribution suggests broad transcriptional remodeling in CLL rather than a unidirectional shift.

Figure 1 highlights the most prominent signals on both sides of the volcano. Among the top upregulated genes, *ABCA6*, *CCDC88A*, *ADTRP*, *TRAC*, *RASGRF1*, *PIGR*, *LEF1*, *LAX1*, *YME1L1*, and *ROR1* exhibited large positive fold changes and extremely strong FDR support, indicating a reproducible CLL-associated expression program. In contrast, the top downregulated genes—*SCN3A*, *IGLC1*, *IGLJ3*, *PARM1*, *EIF2AK3*, *PMEPA1*, *CXorf57*, *CD55*, *SNX22*, and *IGLV1-44*—showed strong negative fold changes with very low FDR values, consistent with marked suppression of specific immune/lineage-linked transcripts and other regulatory components.

These ranked candidates are summarized in Table 2, which lists the top 10 upregulated and top 10 downregulated genes in GSE50006, including their log2FC and adjusted significance. Together, Figure 1 and Table 2 provide a compact overview of both the global DEG landscape and the highest-confidence gene-level signals.

#### 3.1.2. Independent Replication of CLL-Associated DEGs: GSE31048

**Figure 2:**
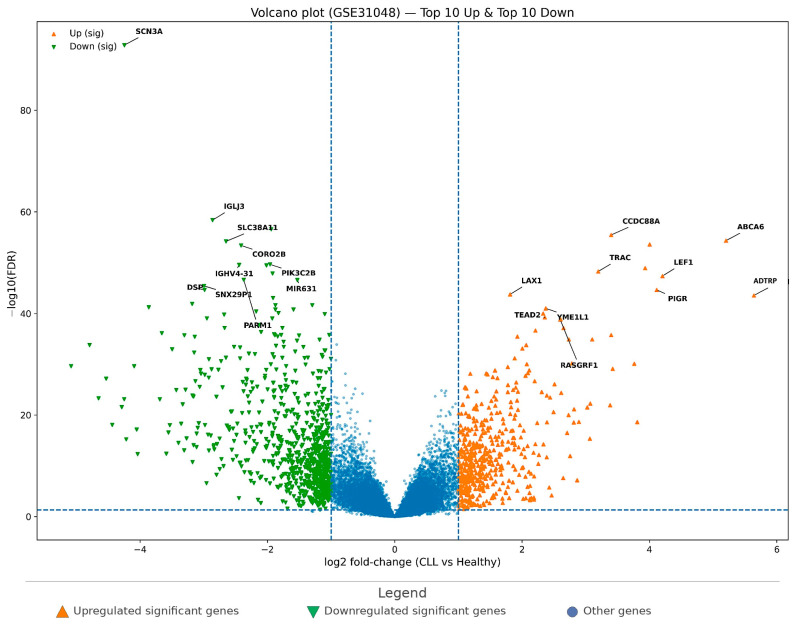
Volcano plot of differential gene expression in GSE31048 (CLL vs. healthy controls). Genes are plotted by log2 fold-change on the *x*-axis and −log10(FDR) on the *y*-axis. Dashed vertical lines indicate the fold-change threshold, and the dashed horizontal line indicates the significance threshold. Orange triangles indicate significantly upregulated genes, and green inverted triangles indicate significantly downregulated genes. Labeled points represent the top-ranked upregulated and downregulated genes selected for downstream comparative analysis.

In the second CLL cohort (GSE31048), the volcano plot (Figure 2) also demonstrates a robust differential expression pattern between CLL and healthy controls. Under the same thresholds (FDR < 0.05 and |log2FC| ≥ 1), 1185 significant genes were detected, including 478 upregulated and 707 downregulated genes. Compared with GSE50006, the total number of significant DEGs was smaller, but the presence of a substantial DEG set supports cohort-level reproducibility.

Importantly, the top-ranked upregulated genes in GSE31048 (Table 3) substantially overlapped with those observed in GSE50006. The leading upregulated candidates included *CCDC88A*, *ABCA6*, *TRAC*, *LEF1*, *PIGR*, *LAX1*, *ADTRP*, *YME1L1*, *TEAD2*, and *RASGRF1*, reinforcing cross-cohort stability of the CLL signature. Similarly, the most prominent downregulated genes included *SCN3A* and *IGLJ3* (also among the strongest downregulated genes in GSE50006), together with additional suppressed transcripts such as *SLC38A11*, *CORO2B*, *PIK3C2B*, *IGHV4-31*, *PARM1*, *MIR631*, *SNX29P1*, and *DSP*.

Figure 2 summarizes the global distribution of DEGs in GSE31048, while Table 3 provides the top 10 up/down ranked list. Collectively, the two CLL datasets support a reproducible CLL-associated transcriptomic pattern, characterized by consistent upregulation of genes such as *CCDC88A*, *ABCA6*, *TRAC*, *LEF1*, *PIGR*, *LAX1*, *ADTRP*, *YME1L1*, *TEAD2*, and *RASGRF1*, together with consistent downregulation signals, including *SCN3A* and immunoglobulin region (locus) transcripts.

### 3.2. Study Selection and Dataset Characteristics for MM (GSE7116, GSE27838)

To define MM-associated expression changes, two GEO datasets were analyzed. GSE7116 included 10 MM samples and 5 healthy controls, while GSE27838 included 8 MM and 8 healthy samples (non-expanded). Key cohort characteristics are summarized in Table 1.

#### 3.2.1. Differential Gene Expression Profile in MM: GSE7116

**Figure 3:**
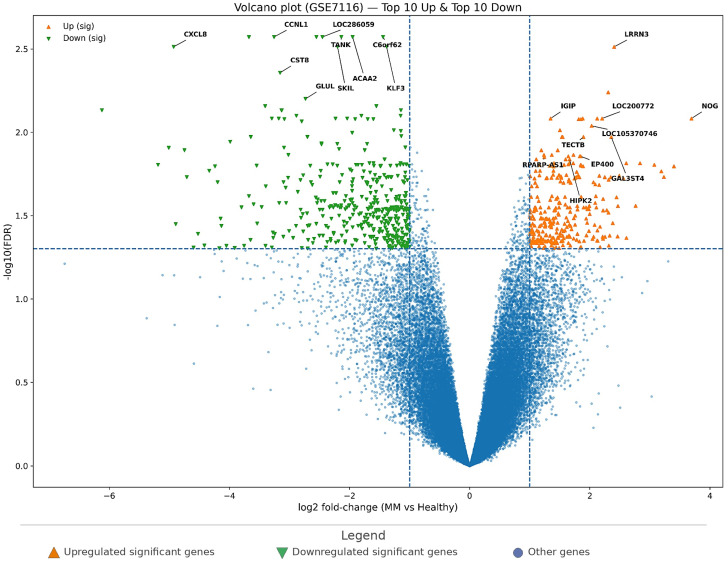
Volcano plot of differential gene expression in GSE7116 (MM vs. healthy controls). Genes are plotted by log2 fold-change on the *x*-axis and −log10(FDR) on the *y*-axis. Dashed vertical lines indicate the fold-change threshold, and the dashed horizontal line indicates the significance threshold. Orange triangles indicate significantly upregulated genes, and green inverted triangles indicate significantly downregulated genes. Labeled points represent the top-ranked upregulated and downregulated genes selected for downstream comparative analysis.

In GSE7116, the volcano plot (Figure 3) demonstrates a discernible MM-associated differential expression pattern. With FDR < 0.05 and |log2FC| ≥ 1, 542 significant genes were identified, including 182 upregulated and 360 downregulated genes, indicating a stronger downregulated component in this dataset.

The top upregulated genes (Table 4) included *LRRN3*, *NOG*, *LOC200772*, *IGIP*, *LOC105370746*, *GAL3ST4*, *TECTB*, *EP400*, *RPARP-AS1*, and *HIPK2*, representing a mixture of coding genes and annotated loci that show strong MM-related activation. Conversely, the top downregulated genes included *CCNL1*, *LOC286059*, *TANK*, *ACAA2*, *C6orf62*, *CXCL8*, *SKIL*, *KLF3*, *CST8*, and *GLUL*, pointing to suppression of immune/inflammatory mediators and metabolic/regulatory transcripts in MM relative to healthy controls.

Together, Figure 3 and Table 4 summarize the MM-associated differential-expression profile in GSE7116 and highlight the strongest ranked candidate genes for downstream interpretation.

#### 3.2.2. Differential Gene Expression Profile in MM: GSE27838 (Non-Expanded)

**Figure 4:**
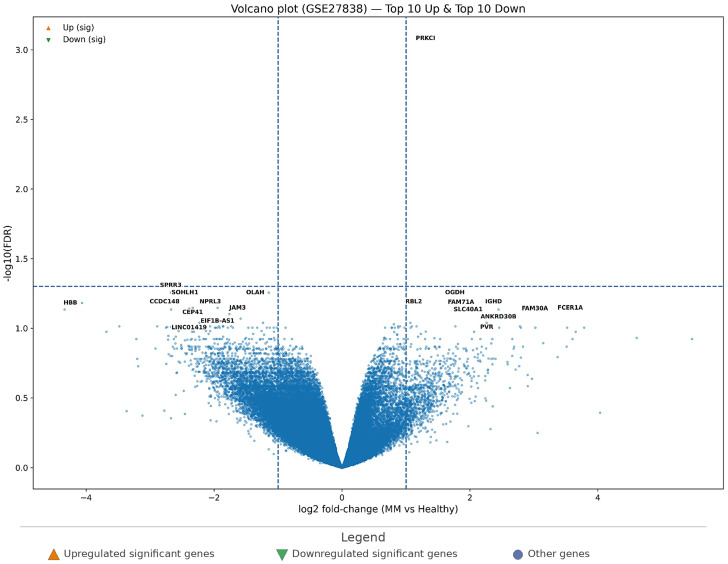
Volcano plot of differential gene expression in GSE27838 (MM vs. healthy controls). Genes are plotted by log2 fold-change on the *x*-axis and −log10(FDR) on the *y*-axis. Dashed vertical lines indicate the fold-change threshold, and the dashed horizontal line indicates the significance threshold. Orange triangles indicate significantly upregulated genes, and green inverted triangles indicate significantly downregulated genes. Labeled points represent the top-ranked upregulated and downregulated genes selected for downstream comparative analysis.

In GSE27838, the volcano plot (Figure 4) was generated with the same thresholds (|log2FC| = 1, FDR = 0.05) to maintain consistency across analyses. However, under these strict criteria, only 1 gene remained significant (*PRKCI*), reflecting limited statistical power and/or greater expression heterogeneity in this cohort under multiple-testing correction. For this reason, while Figure 4 still displays the standard threshold lines for interpretability, the “top genes” were prioritized primarily by adjusted significance ranking to provide a structured candidate list for downstream integrative analyses rather than as definitive FDR-positive biomarkers from this single dataset.

Despite this limitation, the ranked top signals in Table 5 offer biologically informative candidates. The top “upregulated” side included PRKCI as the most prominent signal, followed by genes such as *RBL2*, *OGDH*, *SLC40A1*, *IGHD*, *FCER1A*, *ANKRD30B*, *FAM30A*, *FAM71A*, and *PVR*. The top “downregulated” candidates included *SPRR3*, *SOHLH1*, *OLAH*, *CCDC148*, *NPRL3*, *HBB*, *CEP41*, *EIF1B-AS1*, *JAM3*, and *LINC01419*. These ranked signals suggest that, despite limited formal significance under strict thresholds, GSE27838 still contributes structured candidate genes for cross-dataset prioritization in MM.

Taken together, the MM results indicate that GSE7116 provides a more statistically supported DEG set, while GSE27838 contributes complementary ranked candidates that should be interpreted through cross-dataset consensus and network-based prioritization rather than single-cohort significance alone.

### 3.3. Cross-Dataset Consensus Signatures and Differential Diagnosis Gene Sets

#### 3.3.1. CLL-Consensus Signature (GSE50006 and GSE31048)

A strict direction-aware intersection of significant genes across the two CLL cohorts yielded a CLL-consensus signature comprising 670 genes with concordant directionality. This consensus set included 668 high-confidence genes supported by statistical significance and effect-size thresholds in both datasets, indicating strong reproducibility of the CLL transcriptomic signal across independent cohorts. The full CLL-consensus list (including direction and summary statistics) is provided in Supplementary Table S1.

#### 3.3.2. MM-Consensus Signature (GSE7116 and GSE27838)

A vote-based, direction-aware consensus strategy was applied to MM cohorts due to the limited number of strictly significant genes in GSE27838. This approach yielded an MM-consensus signature comprising 60 genes with concordant directionality across datasets and effect-size support, with statistical significance present in at least one cohort. The full MM-consensus list is provided in Supplementary Table S2.

#### 3.3.3. CLL-Specific, MM-Specific, and Shared Signatures

To support downstream differential diagnosis analyses, disease-specific and shared gene signatures were derived from consensus lists. The shared set contained 5 genes present in both consensus signatures. The CLL-specific set contained 665 genes that were unique to the CLL-consensus signature, whereas the MM-specific set contained 55 genes unique to the MM-consensus signature. These three gene sets constitute the primary inputs for the next phase of the study, including regulatory inference and miRNA target enrichment analysis. The complete lists are provided in Supplementary Table S3.

### 3.4. Experimentally Validated miRNA Target Enrichment (miRTarBase-Weighted)

**Figure 5:**
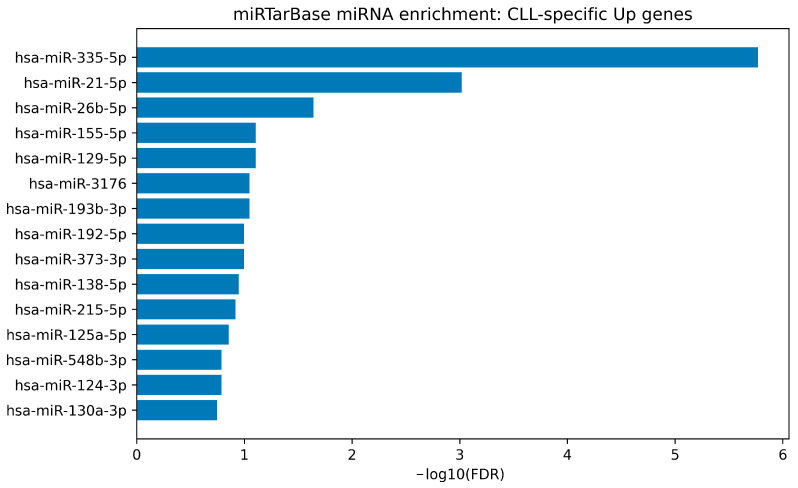
miRTarBase-weighted miRNA enrichment for the CLL-specific up consensus signature. Bar plot shows the top enriched miRNAs whose experimentally validated target genes (MiRTarBase microRNA Targets; Harmonizome) are over-represented among CLL-specific upregulated consensus genes. Bars represent −log10(BH-FDR) from hypergeometric over-representation analysis, and miRNAs are ranked by adjusted significance. Overlap size (k) corresponds to the number of signature genes targeted by each miRNA.

**Figure 6:**
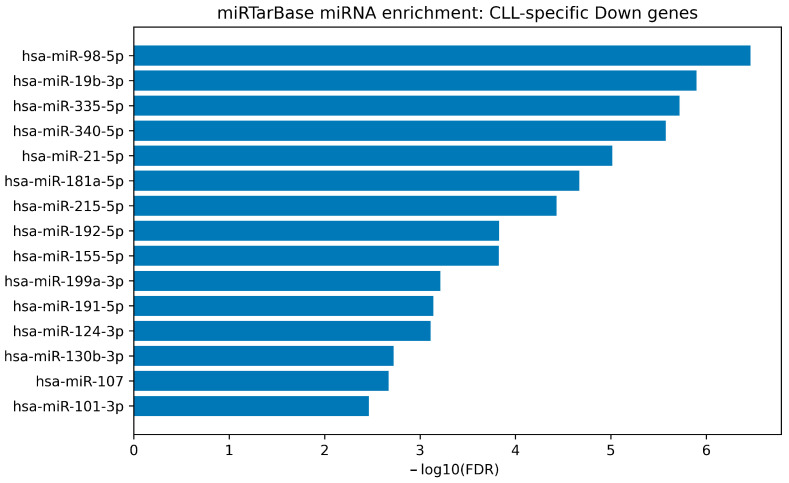
miRTarBase-weighted miRNA enrichment for the CLL-specific down consensus signature. Bar plot summarizes the top enriched miRNAs for the CLL-specific downregulated consensus gene set using MiRTarBase-validated interactions (Harmonizome). Bars represent −log10(BH-FDR) from hypergeometric enrichment, ranked by adjusted *p*-value. The broader enrichment pattern reflects extensive validated miRNA–target coverage across the CLL-down signature.

**Figure 7:**
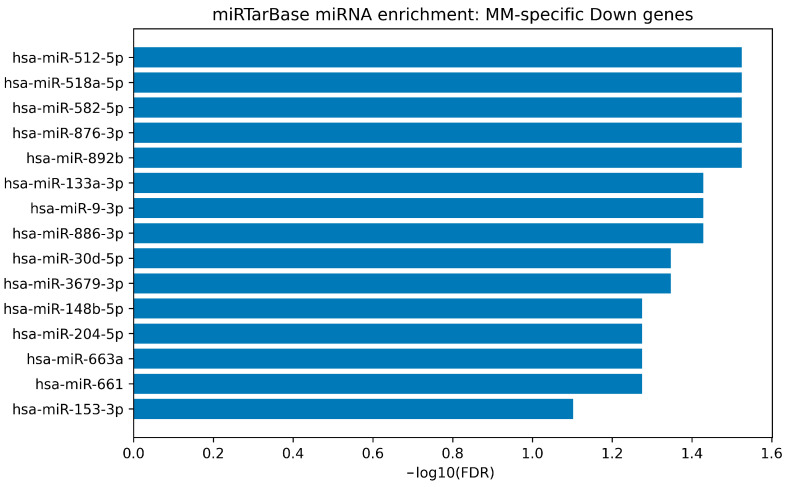
miRTarBase-weighted miRNA enrichment for the MM-specific down consensus signature. Bar plot depicts the top enriched miRNAs whose validated targets are over-represented in the MM-specific downregulated consensus gene set. Bars represent −log10(BH-FDR). Results should be interpreted cautiously when enriched miRNAs correspond to very small target sets in the underlying library, as this may inflate enrichment ratios.

**Figure 8:**
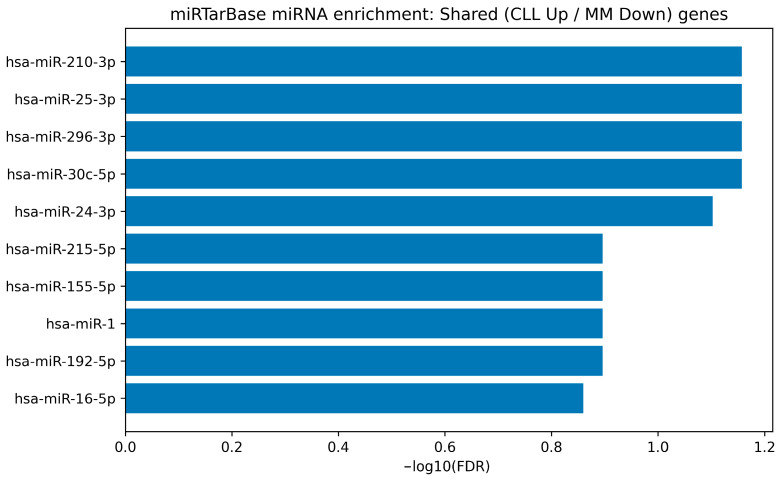
miRTarBase-weighted miRNA enrichment for the discordant shared subset (CLL up/MM down). Bar plot shows the top-ranked miRNAs (by BH-FDR) whose validated targets overlap with the discordant shared genes exhibiting opposite regulation between CLL and MM (CLL up/MM down). Bars represent −log10(BH-FDR). Because this subset contains only a small number of genes, enrichment is underpowered; therefore, miRNAs are presented as differential-diagnosis-relevant candidates rather than definitive regulators.

To translate the consensus-derived gene signatures into an experimentally supported miRNA-regulatory layer, we performed miRNA target enrichment using the MiRTarBase “microRNA Targets” gene-set collection available through Harmonizome. The full enrichment output for each direction-stratified signature is provided in Supplementary Table S4, and the top-ranked miRNAs are visualized as bar plots in Figure 5, Figure 6, Figure 7 and Figure 8.

Within the CLL-specific upregulated consensus gene set, enrichment identified a limited number of statistically supported miRNAs. The strongest signal was hsa-miR-335-5p (FDR = 2.0 × 10^−6^; overlap = 59 targets; enrichment ≈ 2.11), followed by hsa-miR-21-5p (FDR = 9.6 × 10^−4^; enrichment ≈ 3.10) and hsa-miR-26b-5p (FDR = 2.27 × 10^−2^; enrichment ≈ 1.79). These results are visually highlighted in Figure 5, indicating that a small set of experimentally validated miRNAs may account for a substantial fraction of the CLL-up transcriptional program.

In contrast, the CLL-specific downregulated consensus gene set displayed a substantially broader enrichment architecture, with many miRNAs passing FDR < 0.05. The top signals included hsa-miR-98-5p (FDR = 3.44 × 10^−7^; enrichment ≈ 3.75), hsa-miR-19b-3p (FDR = 1.26 × 10^−6^; enrichment ≈ 4.83), hsa-miR-335-5p (FDR = 1.90 × 10^−6^), hsa-miR-340-5p (FDR = 2.66 × 10^−6^; enrichment ≈ 6.70), and hsa-miR-21-5p (FDR = 9.66 × 10^−6^). This enrichment landscape is displayed in Figure 6 and detailed in Supplementary Table S4, suggesting that the downregulated CLL-consensus program overlaps extensively with experimentally validated miRNA target regulons.

For the MM-specific downregulated gene set, enrichment returned multiple miRNAs meeting FDR < 0.05; however, several of these signals corresponded to very small target sets (including entries with a single target gene in the underlying library), which can inflate enrichment ratios and reduce biological interpretability. Accordingly, MM-specific enriched miRNAs were treated as hypothesis-generating candidates rather than definitive regulators. The ranked MM-down enrichment is shown in Figure 7.

For the discordant shared subset (CLL up/MM down), no miRNAs passed the FDR < 0.05 threshold, which is expected given the very small gene-set size (three genes). Nevertheless, the top-ranked candidates by adjusted significance—including hsa-miR-210-3p, hsa-miR-25-3p, hsa-miR-296-3p, hsa-miR-30c-5p, and hsa-miR-24-3p—remain relevant to differential diagnosis because they target genes exhibiting opposite regulation between CLL and MM. These candidates are shown in Figure 8.

### 3.5. miRNA–mRNA Bipartite Networks and Hub miRNA Prioritization

The enrichment and network analysis results were summarized using bar plots of the top enriched miRNAs for each signature, Venn or UpSet-style overlap summaries for CLL-biased and MM-biased miRNAs, and network visualizations of the highest-confidence miRNA–mRNA modules derived from validated miRNA–target interactions. Cytoscape-ready edge and node tables for each network are provided in Supplementary Table S5, and hub miRNAs ranked by degree (i.e., the number of targeted signature genes) are summarized in Supplementary Table S6. Betweenness centrality was examined as a secondary topological descriptor; however, hub prioritization and reporting were based primarily on degree to maintain interpretability across networks. Although degree was used as the primary hub metric for interpretability across networks of different sizes, future analyses should test the stability of top-ranked hubs across additional centrality measures such as eigenvector centrality and closeness-based metrics.

The CLL-specific (up) network formed a compact topology (111 edges; 98 nodes) driven by three retained miRNAs, consistent with the narrow enrichment footprint observed for the CLL-up signature (Supplementary Table S5). This network is visualized in Supplementary Figure S1, where hsa-miR-335-5p is clearly highlighted as the dominant regulator targeting the majority of CLL-up consensus genes. Degree-based hub ranking confirmed hsa-miR-335-5p as the leading miRNA hub (degree = 60) in the CLL-up network (Supplementary Table S6).

In contrast, the CLL-specific (down) network was substantially denser (572 edges; 273 nodes) with 47 miRNAs, mirroring the broader enrichment structure observed for CLL-down (Supplementary Table S5). Due to dense connectivity, a readability-optimized visualization subset is shown in Supplementary Figure S2, while the complete, unfiltered network is fully available in Supplementary Table S5. Hub analysis identified hsa-miR-335-5p as the top regulator (degree = 74), followed by hsa-miR-124-3p (degree = 39), hsa-miR-192-5p (degree = 33), and hsa-miR-98-5p (degree = 29) (Supplementary Table S6), supporting a concentrated set of experimentally validated miRNA regulators aligned with the reproducible downregulated CLL program. The MM-specific (down) network remained small (12 edges; 14 nodes) with 10 retained miRNAs, reflecting the limited MM-specific consensus footprint and the enrichment pattern influenced by small underlying target sets (Supplementary Table S5). The corresponding network structure is shown in Supplementary Figure S3. Consistent with this sparsity, hub miRNAs exhibited low degrees and should be prioritized conservatively (Supplementary Table S6).

Finally, the discordant shared (CLL up/MM down) network contained 10 edges and 12 nodes with 10 miRNAs retained by ranking, providing a focused shortlist of experimentally supported regulatory links directly mapped to genes exhibiting opposite regulation between CLL and MM (Supplementary Table S5). This discordant network is visualized in Supplementary Figure S4 and is particularly relevant to differential diagnosis, although enrichment is underpowered due to the small gene-set size; therefore, these miRNAs should be interpreted as candidates rather than definitive regulators (Supplementary Table S6). Collectively, the network analyses complement the miRTarBase-weighted enrichment results (Figure 5, Figure 6, Figure 7 and Figure 8; Supplementary Table S4) by revealing topology-driven hub miRNAs and interaction-level evidence that support prioritization of candidate miRNA regulators inferred from blood-derived transcriptional signatures in CLL and MM.

## 4. Discussion

Differential diagnosis between CLL and MM can be challenging when relying on nonspecific systemic signals, particularly when clinical presentation overlaps or when peripheral blood findings are confounded by infection, therapy exposure, and immune dysregulation. Although the two diseases arise from distinct B-cell differentiation stages (CLL from mature B lymphocytes and MM from malignant plasma cells within the bone marrow niche), both can imprint measurable transcriptional and post-transcriptional regulatory effects in circulating immune compartments. In this study, we used a conservative, cross-cohort strategy to define direction-consistent disease signatures from peripheral blood-derived transcriptomes and then projected these signatures onto an experimentally supported regulatory layer through miRTarBase-weighted enrichment and miRNA–mRNA bipartite network. This design directly targets robustness and interpretability, prioritizing regulatory candidates supported by curated experimental evidence rather than purely in silico target predictions [22].

From a clinical workflow perspective, the emphasis on cross-cohort reproducibility and mechanistic interpretability is complementary to guideline-based diagnostic frameworks: CLL evaluation integrates persistent B-lymphocytosis with immunophenotyping and standardized response/prognostic criteria, whereas MM diagnosis relies on bone marrow plasma cell assessment and myeloma-defining events—creating a practical rationale for blood-based molecular adjuncts that may be most useful in longitudinal monitoring, in biologically ambiguous peripheral-blood findings, or as complementary molecular support rather than stand-alone diagnostic substitutes [2].

A key observation is the pronounced difference in cross-dataset stability between CLL and MM. The CLL direction-consistent consensus signature was large and showed strong between-cohort agreement, consistent with the biology of CLL as a malignancy characterized by abundant circulating tumor cells and systemic immune remodeling that is readily detectable in peripheral blood expression profiles. In contrast, the MM consensus signature was much smaller and more sensitive to cohort-specific statistical signal, a pattern that is biologically plausible because the dominant tumor burden in MM resides in the bone marrow microenvironment, and peripheral blood gene expression changes can be subtler, more heterogeneous, or driven by shifts in immune subset proportions rather than direct tumor transcript abundance. Contemporary reviews and atlasing studies emphasize that MM pathophysiology is tightly coupled to bone marrow cellular niches and immune states, which may not be fully captured by bulk PBMC/whole-blood measurements alone [23]. Accordingly, MM-focused extensions of this framework will likely benefit from explicitly incorporating bone marrow-centric transcriptomic contexts (tumor–stroma–immune niches) alongside peripheral blood, because key MM regulatory programs can be compartmentalized and under-represented in PBMC-only sampling [24]. The limited number of strictly significant genes in GSE27838 likely reflects a combination of modest sample size, biological heterogeneity, and attenuation of disease-related signal in peripheral blood relative to marrow-centered biology. Accordingly, MM-associated findings in the present study should be interpreted conservatively, and future extensions should incorporate additional GEO cohorts and/or alternative consensus strategies to strengthen MM-specific inference. Future work should compare strict-intersection, vote-based, and rank-based consensus strategies using overlap-based stability metrics to quantify how candidate list size and biological interpretation change across rules.

The miRTarBase-weighted enrichment results provide a biologically meaningful bridge from transcriptomic signatures to candidate circulating miRNA regulators. For the CLL-specific consensus sets, enrichment analysis revealed a compact upregulated miRNA signal and a broader, denser downregulated regulatory landscape, consistent with extensive post-transcriptional control in CLL. These complementary enrichment patterns were consistently observed across the enrichment analyses. This asymmetry is consistent with the notion that CLL is shaped by extensive post-transcriptional regulation and microenvironmental signaling, including B-cell receptor (BCR) activation and cytokine-driven survival pathways, which affect broad gene programs and are frequently regulated through miRNA-mediated mechanisms [25]. Using experimentally curated interaction resources is a defensible design choice here because it prioritizes interactions supported by direct assays and continuous database curation, which can improve biological credibility when translating “gene programs” into candidate miRNA regulators [22].

Across CLL-associated findings, several prioritized miRNAs have strong precedent in lymphoid malignancy biology and blood-based biomarker research. MiR-21-5p emerges as a recurrent CLL-associated candidate regulator and remains biologically plausible in light of its known roles in B-cell survival signaling and hematologic malignancy biology [26]. Likewise, miR-19b-3p, as part of the miR-17-92 cluster family broadly linked to B-cell transformation, supports the plausibility of disease-relevant post-transcriptional control in CLL [27]. The prominence of let-7 family-related signals such as miR-98-5p is also coherent with established roles of let-7-related miRNAs in immune differentiation and cancer-relevant gene regulation [28]. These broader CLL-associated enrichment and network patterns support the biological plausibility of the prioritized CLL miRNA candidates.

The repeated appearance of miR-21 in hematologic malignancy biomarker studies strengthens its plausibility as a “cross-platform” candidate, but also highlights the need to test specificity against inflammatory confounding in real-world blood samples [29]. Similarly, signals mapping to the miR-17-92 family are mechanistically coherent in B-cell malignancy contexts, given extensive evidence linking this cluster family to B-cell transformation and regulatory control of proliferative programs [30]. Let-7 family biology provides a plausible conceptual bridge between immune-state remodeling and cancer-relevant post-transcriptional control, supporting the interpretability of let-7-related enrichment signals in peripheral blood signatures [31].

In the CLL-down network, hub structure further prioritized miRNAs with broad experimentally validated target connectivity within the consensus gene space, suggesting that regulatory reach may be more important than single-gene effects when designing panels for differential diagnosis. This broader hub-centered pattern is reflected in the corresponding supplementary network summaries (Supplementary Figure S2; Supplementary Table S6).

For MM, the miRNA enrichment of the MM-specific downregulated consensus set produced several nominally significant signals but also displayed patterns consistent with small-set and target-universe artifacts, which is an important caution for biomarker interpretation. When gene lists are small, over-representation statistics can inflate enrichment ratios, and ranking may be disproportionately influenced by a few curated interactions rather than a stable distributed signal. This supports treating MM-specific miRNA candidates from the current PBMC-driven consensus as hypothesis-generating until corroborated by independent MM cohorts and/or bone marrow-derived transcriptional signatures [32]. Notably, several miRNAs often discussed in the MM biomarker literature, including miR-21 and miR-192, have reported associations with MM in peripheral blood or serum studies, indicating that convergence between transcriptome-projected regulators and empirically measured circulating miRNAs remains biologically plausible even if the present MM consensus signal is conservative [33]. Because ORA-style enrichment is sensitive to gene-set size and background definitions, reporting and interpretation benefit from explicitly documenting universe choices and complementing ORA outputs with stability checks (e.g., resampling or rank-based approaches) when signatures are small [34].

A particularly diagnosis-relevant component of the design is the shared-gene intersection analysis, where shared genes were stratified by directionality to distinguish concordant disease-vs-control effects from discordant disease-biased regulation. The discordant shared subset (CLL up/MM down) is conceptually attractive for differential diagnosis because it can encode “opposite-direction” molecular behavior rather than generic inflammation or stress responses. Even though enrichment was underpowered for this small subset, the candidate miRNAs nominated in this context remain valuable as a focused shortlist for downstream prioritization—especially if these miRNAs are detectable and stable in plasma/exosomes and if their targets participate in immune tolerance, autophagy, and cytokine signaling. In this regard, genes such as CEACAM1 and SOCS3 provide mechanistic anchors linking disease-specific immune states to plausible miRNA-mediated regulation, reinforcing why discordant-direction features may add discriminative value beyond simple case–control signatures [35]. Taken together, these discordant-direction features support a focused shortlist of candidate regulators with potential differential-diagnostic relevance.

Network topology adds an additional layer of prioritization by identifying miRNAs that act as hubs across multiple consensus targets, which can be advantageous for diagnostic panel design because hubs may provide more stable readouts across cohorts and measurement platforms. In the present study, CLL-specific networks showed clear differences in density between upregulated and downregulated signatures, whereas the MM-specific down network remained sparse, consistent with the conservative MM consensus size and the need for cautious interpretation. This pattern further suggests that MM prioritization may benefit from additional PBMC/whole-blood cohorts, incorporation of bone marrow microenvironment transcriptomics, and direct integration with circulating miRNA datasets to test whether transcriptome-projected hubs are measurably perturbed in biofluids [36].

Several limitations should be emphasized to frame biomarker-related claims appropriately. First, PBMC/whole-blood expression profiles reflect both disease biology and shifts in immune cell composition; without cell-type deconvolution or single-cell validation, some gene-level differences may reflect proportional changes rather than within-cell transcriptional rewiring. Because the present framework was built on harmonized cross-cohort differential-expression summaries rather than uniformly reprocessed raw cohort matrices suitable for deconvolution pipelines, formal cell-type deconvolution was not implemented in this version. Future work should explicitly test whether prioritized signature genes and hub miRNAs remain informative after accounting for inferred leukocyte fractions. Second, miRTarBase-weighted enrichment prioritizes experimentally validated interactions, which strengthens biological credibility but also introduces literature and context bias: well-studied miRNAs and widely validated targets may be favored independent of the specific biological compartment under study (e.g., blood, bone marrow, or tumor tissue) [22]. Third, projecting PBMC/whole-blood-derived gene signatures onto validated miRNA–target interaction spaces provides a biologically informed framework for prioritizing candidate regulators of blood transcriptional programs, but does not constitute direct evidence of circulating biomarker performance. Therefore, any liquid-biopsy or circulating-miRNA interpretation requires independent validation in plasma-, serum-, or exosome-based cohorts before clinical diagnostic claims can be made [37]. Accordingly, the present results should be interpreted as a prioritization framework for candidate miRNA regulators inferred from blood-derived transcriptional programs, rather than as direct evidence of circulating biomarker performance. Reproducibility in the present study should therefore be interpreted as cross-cohort concordance under a predefined harmonized thresholding scheme, rather than as demonstrated stability across multiple parameter settings. A full reanalysis from raw CEL/FASTQ files or implementation of a formal rank-based meta-analysis strategy may further improve cross-cohort comparability and represents an important direction for future work.

Given the strong dependence of bulk-blood profiles on leukocyte mixtures, incorporating computational deconvolution (or, at a minimum, sensitivity analyses) can help separate “composition” from “within-cell” regulation and sharpen biological interpretation of consensus signatures [38]. For downstream wet-lab validation, pre-analytical control is critical: hemolysis can inflate canonical circulating miRNAs (including commonly used normalizers such as miR-16), and qPCR reporting/normalization should follow established minimum-information standards to improve cross-lab reproducibility [39]. At a minimum, future plasma/serum validation studies should document hemolysis assessment, sample processing time and storage conditions, exclusion or flagging of cellular contamination, and justification of normalization strategy (e.g., spike-in controls and/or empirically stable endogenous references) in line with MIQE-oriented reporting principles. These measures are essential to distinguish true extracellular miRNA signals from artifacts introduced by sample handling or blood-cell contamination.

Despite these constraints, the analysis provides a structured, reproducible route from cross-cohort transcriptomic signatures to experimentally supported miRNA regulators and interpretable hub networks. Importantly, the study yields three diagnostically relevant outputs: robust disease-associated consensus gene programs, experimentally supported miRNA candidate shortlists, and interpretable hub-centered regulatory networks. Together, these outputs define a prioritized candidate feature space for future diagnostic modeling, rather than a completed predictive framework with quantified performance metrics. Future work should focus on independent external validation cohorts, harmonized preprocessing/meta-analysis across additional GSE studies, and integration with directly measured circulating miRNA datasets to determine whether top-ranked hubs (e.g., miR-21-5p and other signature-specific candidates) retain discriminative value across laboratories and specimen handling conditions.

## 5. Conclusions

In this in silico study, direction-consistent consensus gene signatures derived from independent GEO cohorts were used to identify robust, disease-biased transcriptional programs in CLL and MM and to translate these programs into an experimentally supported miRNA regulatory layer using miRTarBase evidence. The workflow prioritized reproducibility across cohorts and enabled partitioning into CLL-specific, MM-specific, and shared/discordant components with direct relevance to differential diagnosis. miRTarBase-weighted enrichment and miRNA–mRNA network topology provided ranked miRNA candidates and hub regulators that constitute a focused shortlist for downstream validation. Overall, these results support a reproducible gene-to-miRNA prioritization framework and motivate independent confirmation in circulating miRNA cohorts and future classifier benchmarking using the prioritized gene/miRNA feature space.

## Figures and Tables

**Table 1:** Summary characteristics of GEO cohorts included in the study.

Dataset	Disease	Cases (n)	Controls (n)	Sample Type	Platform	Notes
GSE50006	CLL	5	2	Peripheral blood CD19+ B cells	Affymetrix microarray	Independent CLL validation cohort; processed data used
GSE31048	CLL	179	33	Peripheral blood/B-cell profile	Affymetrix microarray	Higher-powered CLL cohort used for cross-cohort consensus
GSE7116	MM	10	5	Peripheral blood mononuclear cells (PBMCs)	Affymetrix microarray	MM cohort with clearer DEG signal
GSE27838 (non-expanded)	MM	8	8	Non-expanded NK-cell profile from peripheral blood	Affymetrix microarray	Used as a conservative MM comparator subset

Notes: Sample sizes and sample-type descriptions were compiled from the corresponding GEO series records and dataset descriptions used in this study. GSE27838 refers to the non-expanded subset analyzed in the present work.

**Table 2:** Top 10 upregulated and top 10 downregulated genes in GSE50006 (CLL vs. Healthy), ranked by FDR.

Gene	log2FC	FDR (adj. P)	*p*. Value	Gene Title
*ABCA6*	5.805	1.43 × 10^−64^	2.62 × 10^−69^	ATP-binding cassette subfamily A member 6
*CCDC88A*	3.661	5.79 × 10^−59^	2.12 × 10^−63^	coiled-coil domain containing 88A
*ADTRP*	5.785	3.42 × 10^−53^	2.50 × 10^−57^	androgen-dependent TFPI-regulating protein
*TRAC*	3.385	6.13 × 10^−51^	7.85 × 10^−55^	T-cell receptor alpha constant
*RASGRF1*	4.484	6.75 × 10^−49^	1.11 × 10^−52^	Ras protein-specific guanine nucleotide releaser factor 1
*PIGR*	4.855	1.34 × 10^−48^	2.44 × 10^−52^	polymeric immunoglobulin receptor
*LEF1*	3.942	5.66 × 10^−48^	1.24 × 10^−51^	lymphoid enhancer binding factor 1
*LAX1*	1.648	7.37 × 10^−47^	2.02 × 10^−50^	lymphocyte transmembrane adaptor 1
*YME1L1*	3.562	9.83 × 10^−46^	2.88 × 10^−49^	YME1-like 1 ATPase
*ROR1*	4.528	2.59 × 10^−44^	8.05 × 10^−48^	receptor tyrosine kinase-like orphan receptor 1
*SCN3A*	−6.473	7.48 × 10^−57^	4.10 × 10^−61^	sodium voltage-gated channel alpha subunit 3
*IGLC1*	−5.076	4.52 × 10^−53^	4.13 × 10^−57^	immunoglobulin lambda constant 1
*IGLJ3*	−5.795	7.37 × 10^−47^	2.01 × 10^−50^	immunoglobulin lambda joining 3
*PARM1*	−4.918	1.10 × 10^−42^	4.21 × 10^−46^	prostate androgen-regulated mucin-like protein 1
*EIF2AK3*	−2.609	5.99 × 10^−41^	2.85 × 10^−44^	eukaryotic translation initiation factor 2 alpha kinase 3
*PMEPA1*	−5.176	7.64 × 10^−41^	4.05 × 10^−44^	prostate transmembrane protein, androgen induced 1
*CXorf57*	−4.722	1.97 × 10^−39^	1.31 × 10^−42^	chromosome X open reading frame 57
*CD55*	−1.243	2.69 × 10^−39^	1.87 × 10^−42^	CD55 molecule (Cromer blood group)
*SNX22*	−2.446	3.94 × 10^−39^	2.81 × 10^−42^	sorting nexin 22
*IGLV1-44*	−5.958	6.45 × 10^−38^	4.83 × 10^−41^	immunoglobulin lambda variable 1–44

Notes: log2FC values are reported for CLL relative to healthy controls; positive values indicate upregulation in CLL. Genes were selected among significant results (FDR < 0.05 and |log2FC| ≥ 1) and filtered to retain unique valid gene symbols; probe-like entries (e.g., “*_at”) and duplicate symbols were excluded.

**Table 3:** Top 10 upregulated and top 10 downregulated genes in GSE31048 (CLL vs. Healthy), ranked by FDR.

Gene	log2FC	FDR (adj.P)	*p*.Value	Gene Title
*CCDC88A*	3.398	3.46 × 10^−56^	2.53 × 10^−60^	coiled-coil domain containing 88A
*ABCA6*	5.202	4.51 × 10^−55^	4.13 × 10^−59^	ATP-binding cassette subfamily A member 6
*TRAC*	3.195	5.36 × 10^−49^	1.27 × 10^−52^	T-cell receptor alpha constant
*LEF1*	4.204	4.36 × 10^−48^	1.2 × 10^−51^	lymphoid enhancer binding factor 1
*PIGR*	4.112	2.29 × 10^−45^	8.39 × 10^−49^	polymeric immunoglobulin receptor
*LAX1*	1.810	1.74 × 10^−44^	6.66 × 10^−48^	lymphocyte transmembrane adaptor 1
*ADTRP*	5.641	2.76 × 10^−44^	1.11 × 10^−47^	androgen-dependent TFPI-regulating protein
*YME1L1*	2.3708	9.79 × 10^−42^	5.01 × 10^−45^	YME1-like 1 ATPase
*TEAD2*	2.356	5.86 × 10^−40^	3.86 × 10^−43^	TEA domain transcription factor 2
*RASGRF1*	2.598	1.65 × 10^−39^	1.21 × 10^−42^	Ras protein-specific guanine nucleotide-releasing factor 1
*SCN3A*	−4.248	1.51 × 10^−93^	2.77 × 10^−98^	sodium voltage-gated channel alpha subunit 3
*IGLJ3*	−2.861	4.07 × 10^−59^	1.49 × 10^−63^	immunoglobulin lambda joining 3
*SLC38A11*	−2.651	6.37 × 10^−55^	6.99 × 10^−59^	solute carrier family 38 member 11
*CORO2B*	−2.414	4.08 × 10^−54^	5.97 × 10^−58^	coronin 2B
*PIK3C2B*	−1.959	2.12 × 10^−50^	3.49 × 10^−54^	phosphatidylinositol-4-phosphate 3-kinase catalytic subunit type 2 beta
*IGHV4-31*	−2.445	2.77 × 10^−50^	5.06 × 10^−54^	immunoglobulin heavy variable 4–31
*PARM1*	−2.372	2.29 × 10^−47^	6.7 × 10^−51^	prostate androgen-regulated mucin-like protein 1
*MIR631*	−1.530	2.65 × 10^−47^	8.25 × 10^−51^	microRNA 631
*SNX29P1*	−2.997	3.66 × 10^−46^	1.2 × 10^−49^	sorting nexin 29 pseudogene 1
*DSP*	−2.988	2.28 × 10^−45^	7.94 × 10^−49^	desmoplakin

Notes: log2FC was calculated as CLL relative to healthy controls; positive values indicate upregulation in CLL. Genes were selected among significant results (FDR < 0.05; |log2FC| ≥ 1, when applicable) and filtered to retain unique valid gene symbols; probe-like entries (e.g., “*_at”) and duplicate symbols were excluded.

**Table 4:** Top 10 upregulated and top 10 downregulated genes in GSE7116 (MM vs. Healthy), ranked by FDR.

Gene	log2FC	FDR (adj. P)	*p*. Value	Gene Title
*LRRN3*	2.4062	0.00307	0.00000048	leucine rich repeat neuronal 3
*NOG*	3.692941	0.00827	0.00000401	noggin
*LOC200772*	2.204969	0.00827	0.0000046	uncharacterized LOC200772
*IGIP*	1.346284	0.00827	0.0000039	IgA-inducing protein
*LOC105370746*	2.031239	0.00914	0.00000686	uncharacterized LOC105370746
*GAL3ST4*	2.35764	0.01066	0.0000093	galactose-3-O-sulfotransferase 4
*TECTB*	1.895368	0.01066	0.0000101	tectorin beta
*EP400*	1.849215	0.01393	0.0000189	E1A binding protein p400
*RPARP-AS1*	1.241928	0.01406	0.0000193	RPARP antisense RNA 1
*HIPK2*	1.663838	0.01451	0.0000207	homeodomain interacting protein kinase 2
*CCNL1*	−3.25661	0.00268	0.000000297	cyclin L1
*LOC286059*	−2.4506	0.00268	0.000000206	tumor necrosis factor receptor superfamily member 10d. decoy with truncated death domain pseudogene
*TANK*	−2.13701	0.00268	0.0000003	TRAF family member associated NFKB activator
*ACAA2*	−1.94937	0.00268	0.000000153	acetyl-CoA acyltransferase 2
*C6orf62*	−1.4424	0.00268	0.000000343	chromosome 6 open reading frame 62
*CXCL8*	−4.9294	0.00307	0.000000618	C-X-C motif chemokine ligand 8
*SKIL*	−2.19999	0.00307	0.000000566	SKI-like proto-oncogene
*KLF3*	−1.38203	0.00307	0.000000505	Kruppel-like factor 3
*CST8*	−3.15706	0.00439	0.000000964	cystatin 8
*GLUL*	−2.73383	0.00629	0.00000161	glutamate-ammonia ligase

Notes: log2FC was calculated as MM relative to healthy controls; positive values indicate upregulation in MM. Genes were selected among significant or top-ranked results after filtering to retain unique valid gene symbols; probe-like entries (e.g., “*_at”) and duplicate symbols were excluded. Annotated LOC entries and other non-coding or less-characterized loci were retained when they represented valid unique annotations in the processed source tables and should be interpreted cautiously.

**Table 5:** Top 10 upregulated and top 10 downregulated genes in GSE27838 (MM vs. Healthy), ranked by FDR.

Gene	log2FC	FDR (adj. P)	*p*. Value	Gene Title
*PRKCI*	1.305649	0.000826	1.51 × 10^−8^	protein kinase C iota
*OGDH*	1.76606	0.055421	4.03 × 10^−6^	oxoglutarate dehydrogenase
*IGHD*	2.371367	0.064423	1.18 × 10^−5^	immunoglobulin heavy constant delta
*RBL2*	1.121092	0.064423	9.11 × 10^−6^	RB transcriptional corepressor like 2
*FCER1A*	3.568011	0.07136	1.74 × 10^−5^	Fc fragment of IgE receptor Ia
*FAM30A*	3.015036	0.072196	2.51 × 10^−5^	family with sequence similarity 30, member A
*ANKRD30B*	2.447083	0.073376	3.22 × 10^−5^	ankyrin repeat domain 30B
*SLC40A1*	1.971986	0.073376	3.22 × 10^−5^	solute carrier family 40 member 1
*FAM71A*	1.861581	0.073376	3.06 × 10^−5^	family with sequence similarity 71 member A
*PVR*	2.262421	0.091305	5.01 × 10^−5^	poliovirus receptor
*SPRR3*	−2.67678	0.055421	5.58 × 10^−6^	small proline rich protein 3
*SOHLH1*	−2.45603	0.055421	2.11 × 10^−6^	spermatogenesis and oogenesis specific basic helix-loop-helix 1
*OLAH*	−1.35534	0.055421	6.08 × 10^−6^	oleoyl-ACP hydrolase
*CCDC148*	−2.77283	0.064423	1.16 × 10^−5^	coiled-coil domain containing 148
*NPRL3*	−2.05894	0.064423	1.18 × 10^−5^	NPR3 like, GATOR1 complex subunit
*HBB*	−4.24573	0.065746	1.44 × 10^−5^	hemoglobin subunit beta
*CEP41*	−2.33361	0.07136	1.97 × 10^−5^	centrosomal protein 41
*EIF1B-AS1*	−1.94476	0.07136	2.09 × 10^−5^	EIF1B antisense RNA 1
*JAM3*	−1.63153	0.07136	1.88 × 10^−5^	junctional adhesion molecule 3
*LINC01419*	−2.38925	0.072196	0.000024	long intergenic non-protein coding RNA 1419

## Data Availability

The original contributions presented in this study are included in the article and Supplementary Material. Further inquiries can be directed to the corresponding author.

## Supplementary Materials

The following supporting information can be downloaded at https://www.mdpi.com/article/10.3390/cimb48040352/s1.
