# Supplementary figures and images for: Candidate miRNA Regulators of Blood Transcriptional Signatures for Differential Diagnosis of Chronic Lymphocytic Leukemia and Multiple Myeloma: A Comprehensive In Silico Study
Source: Curr Issues Mol Biol. 2026 Mar 27;48(4):352. doi: 10.3390/cimb48040352 (PMC13114804; doi:10.3390/cimb48040352)

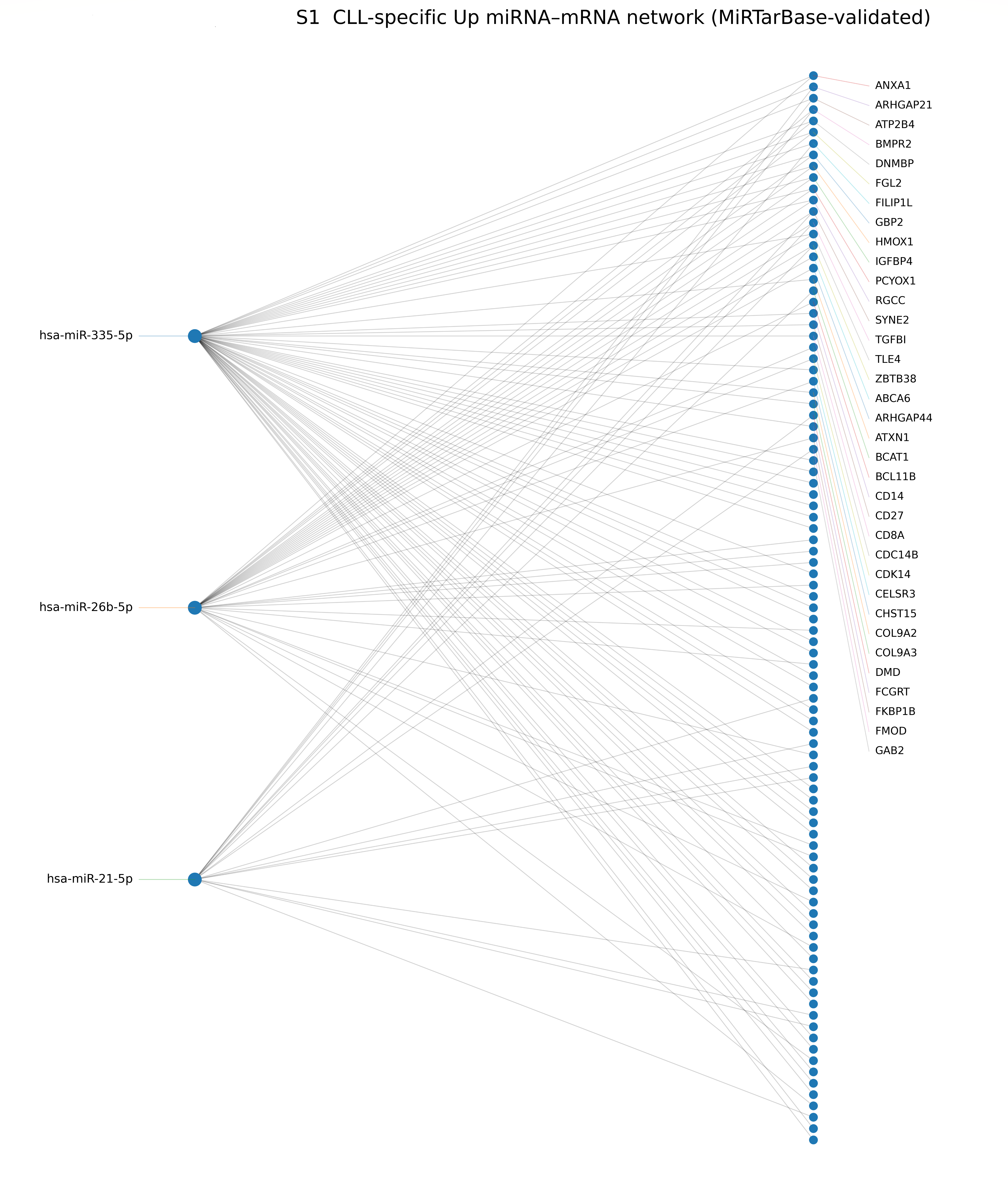

Supplement: Supplementary file 1 [file cimb-48-00352-s001.zip › Supplementary Figure S1.png]

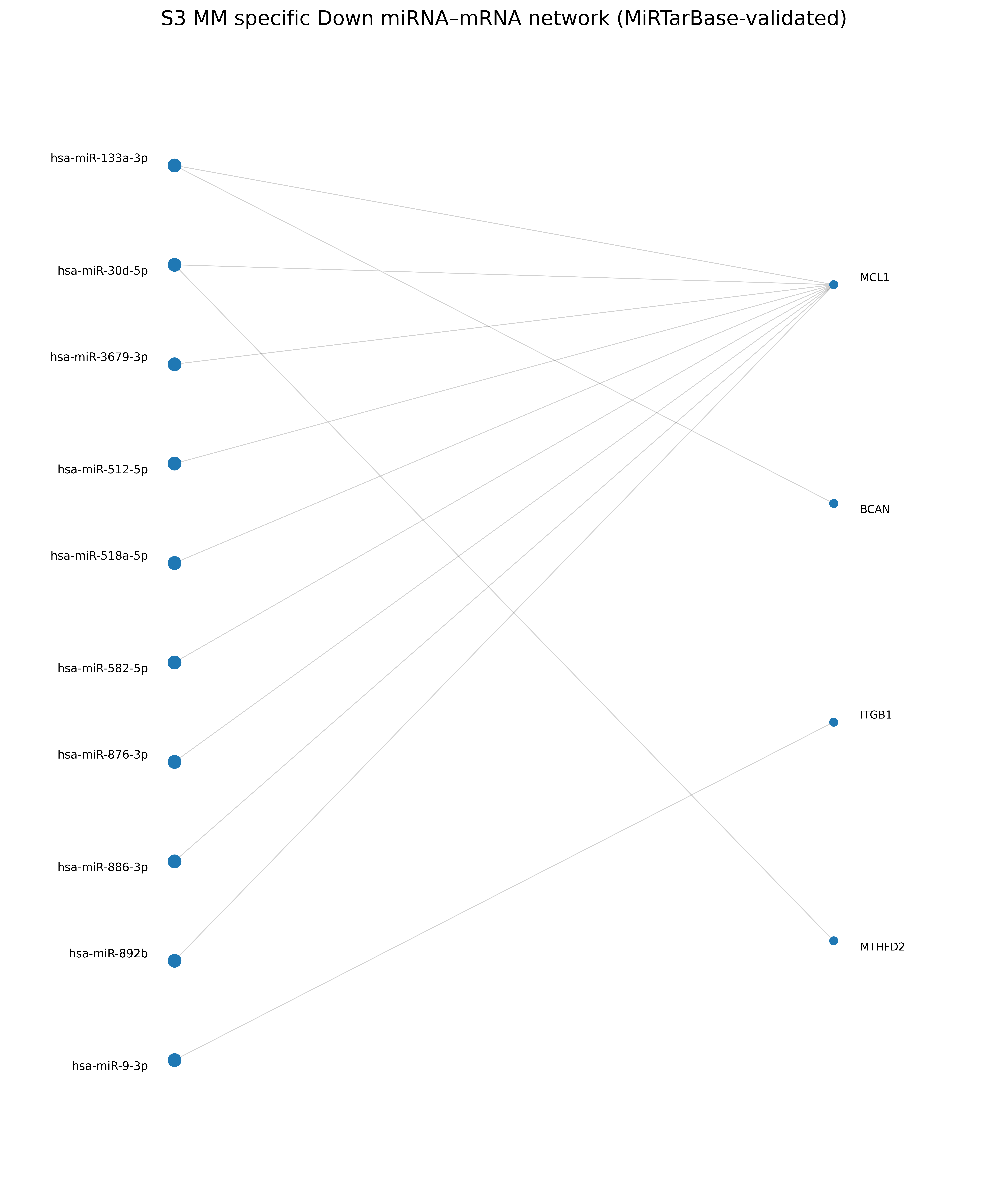

Supplement: Supplementary file 1 [file cimb-48-00352-s001.zip › Supplementary Figure S3.png]

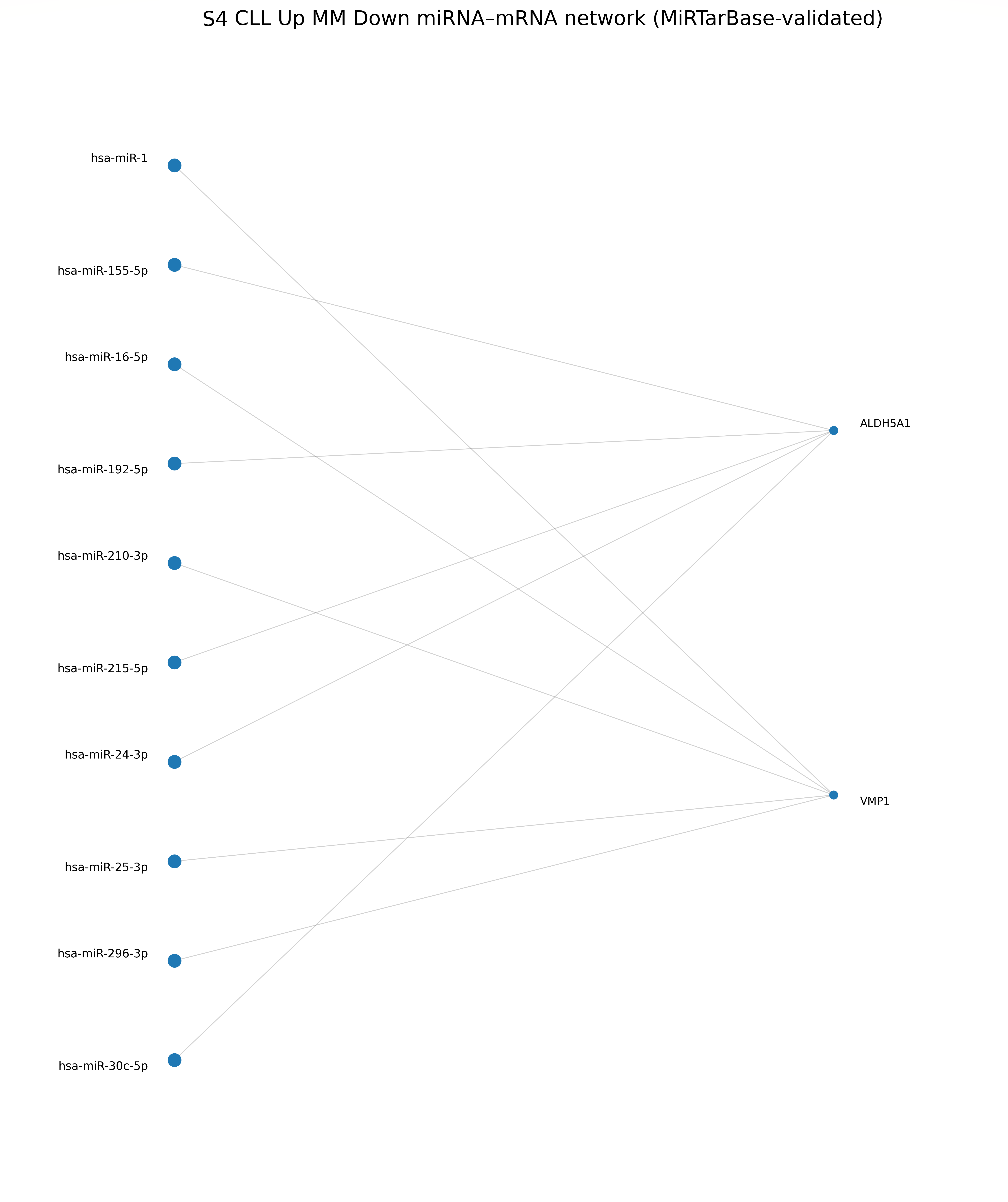

Supplement: Supplementary file 1 [file cimb-48-00352-s001.zip › Supplementary Figure S4 .png]
